# Age interferes with sensorimotor timing and error correction in the supra-second range

**DOI:** 10.3389/fnagi.2022.1048610

**Published:** 2023-01-10

**Authors:** Bettina Pollok, Amelie Hagedorn, Vanessa Krause, Sonja A. Kotz

**Affiliations:** ^1^Institute of Clinical Neuroscience and Medical Psychology, Medical Faculty, University Hospital Düsseldorf, Heinrich Heine University Düsseldorf, Düsseldorf, Germany; ^2^Department of Neuropsychology and Psychopharmacology, Faculty of Psychology and Neuroscience, Maastricht University, Maastricht, Netherlands; ^3^Department of Neuropsychology, Mauritius Hospital and Neurorehabilitation Center Meerbusch, Meerbusch, Germany

**Keywords:** healthy, sensorimotor, sensorimotor synchronization, sub-second and supra-second, tapping, timing

## Abstract

**Introduction:**

Precise motor timing including the ability to adjust movements after changes in the environment is fundamental to many daily activities. Sensorimotor timing in the sub-and supra-second range might rely on at least partially distinct brain networks, with the latter including the basal ganglia (BG) and the prefrontal cortex (PFC). Since both structures are particularly vulnerable to age-related decline, the present study investigated whether age might distinctively affect sensorimotor timing and error correction in the supra-second range.

**Methods:**

A total of 50 healthy right-handed volunteers with 22 older (age range: 50–60 years) and 28 younger (age range: 20–36 years) participants synchronized the tap-onsets of their right index finger with an isochronous auditory pacing signal. Stimulus onset asynchronies were either 900 or 1,600 ms. Positive or negative step-changes that were perceivable or non-perceivable were occasionally interspersed to the fixed intervals to induce error correction. A simple reaction time task served as control condition.

**Results and Discussion:**

In line with our hypothesis, synchronization variability in trials with supra-second intervals was larger in the older group. While reaction times were not affected by age, the mean negative asynchrony was significantly smaller in the elderly in trials with positive step-changes, suggesting more pronounced tolerance of positive deviations at older age. The analysis of error correction by means of the phase correction response (PCR) suggests reduced error correction in the older group. This effect emerged in trials with supra-second intervals and large positive step-changes, only. Overall, these results support the hypothesis that sensorimotor synchronization in the sub-second range is maintained but synchronization accuracy and error correction in the supra-second range is reduced in the elderly as early as in the fifth decade of life suggesting that these measures are suitable for the early detection of age-related changes of the motor system.

## Introduction

Precise motor timing as well as the ability to adapt one’s own movements when changes in the environment occur, represent fundamental prerequisites for the successful interaction with our physical and social environment. Movements, speech production as well as comprehension are based on precise timing in the millisecond range (e.g., Kotz and Schwartze, 2010; Petter et al., 2016). Perceptual and motor timing has been related to a cerebello-basal ganglia-cortical network (for reviews see Lewis and Miall, 2003; Rubia and Smith, 2004; Buhusi and Meck, 2005; Witt et al., 2008; Wiener et al., 2010; Grahn, 2012; Merchant et al., 2013; Petter et al., 2016). On a cortical level, primary motor and premotor cortices (M1/PMC), the supplementary motor area (SMA), the parietal cortex, and several regions of the prefrontal cortex have been identified as key areas for the temporally precise execution of movements (for reviews see Lewis and Miall, 2003; Rubia and Smith, 2004; Buhusi and Meck, 2005; Witt et al., 2008; Wiener et al., 2010; Grahn, 2012; Merchant et al., 2013; Petter et al., 2016). Timing in the sub-and supra-second range may involve at least partially distinct networks (for reviews see Lewis and Miall, 2003; Wiener et al., 2010; Grahn, 2012). Along these lines, the cerebellum and related motor areas likely operate on time intervals in the millisecond range [e.g., (Schwartze et al., 2016); for reviews see (Ivry, 1996; Lewis and Miall, 2003; Buhusi and Meck, 2005; Wiener et al., 2010)]. In contrast to this, the basal ganglia (BG) and a right-hemispheric fronto-parietal network are involved in estimating time intervals in the supra-second range as suggested by imaging (Macar et al., 2002), clinical (Mangels et al., 1998), and non-invasive brain stimulation data (Koch et al., 2003; Jones et al., 2004); for reviews see (Lewis and Miall, 2003; Rubia and Smith, 2004; Buhusi and Meck, 2005; Wiener et al., 2010). These findings suggest that timing in the sub-second range is controlled by an involuntary, non-attentional timing system, while timing in the supra-second range seems to be related to a voluntary attention-related system (for reviews see Lewis and Miall, 2003; Buhusi and Meck, 2005; Wiener et al., 2010). Hence, the proposed brain networks distinctively involved in sub-and supra-second timing may reflect different attentional and memory demands in respective tasks (Rammsayer and Ulrich, 2005).

The flexible adjustment of one’s own movements to changes in the environment requires precise performance monitoring as well as the efficient correction of subsequent actions. Movements can be adapted even to small temporal changes that were not consciously perceived (for reviews see Repp, 2005; Repp and Su, 2013). In contrast to this, conscious error correction depends on attention and awareness of the tempo-change (for reviews see Repp, 2005; Repp and Su, 2013). While the network subserving non-conscious error correction is less clear, conscious error correction has been related to the cerebellum – in particular the left posterior cerebellar lobe – (Stephan et al., 2002; Thaut et al., 2009; Bijsterbosch et al., 2011b), prefrontal (Stephan et al., 2002; Thaut et al., 2009; Bijsterbosch et al., 2011b), premotor (Stephan et al., 2002; Bijsterbosch et al., 2011a), inferior parietal (Thaut et al., 2009; Bijsterbosch et al., 2011b), and anterior cingulate cortices (ACC; Stephan et al., 2002; Jantzen et al., 2018). Stephan et al. (2002) concluded that fully conscious adaptation to changes of an external pacing signal involves the ACC as well as the dorsolateral prefrontal cortex (DPFC). Findings from a patient study additionally suggest reduced attention-dependent error correction in patients with BG-dysfunction (Schwartze et al., 2011).

Normal aging is associated with grey as well as white matter atrophy, particularly affecting frontal brain volume (Raz et al., 1997; Jernigan et al., 2001; Raz et al., 2005); for reviews see (Seidler et al., 2010; Lockhart et al., 2014) and BG structures (e.g., Raz et al., 1995; Jernigan et al., 2001; Fjell et al., 2017). While grey matter volume loss becomes evident already prior to the age of 50 (Lockhart et al., 2014), cerebral white matter volume remains relatively stable until the age of 70 (Jernigan et al., 2001); for a review see (Seidler et al., 2010). Next to these structural alterations, functional changes as indicated by reduced task-related activation of the DPFC (Grady et al., 2006) and reduced functional connectivity [for a review see (Damoiseaux, 2017)] affecting frontal and parietal areas were found (e.g., Hrybouski et al., 2021). Moreover, older adults recruit larger networks including the prefrontal cortex when performing basic motor tasks suggesting a shift from automatic to attentional motor control with increasing age (for a review see Seidler et al., 2010).

Previous studies suggest that the prefrontal cortex as well as the BG are particularly vulnerable to age-related changes (Raz et al., 1995, 1997, 2005; Jernigan et al., 2001; Fjell et al., 2017); for reviews see (Seidler et al., 2010; Lockhart et al., 2014). Since timing in the supra-second range as well as conscious error correction have been related to a fronto-parietal network, the present study aimed at investigating the effect of normal aging on motor-timing in the sub-and supra-second range including error correction to unpredictable step-changes of the pacing signal. We hypothesized reduced synchronization accuracy in trials with supra-second intervals and reduced error correction in trials with large (i.e., perceivable) step-changes, interspersed in a sequence of an otherwise regularly presented auditory pacing signal.

## Materials and methods

### Participants

Fifty-one healthy right-handed volunteers were recruited. Handedness was checked with the Edinburgh Handedness Inventory (EHI; Oldfield, 1971). The individual health condition was determined by the participants’ self-reports. None of them reported impaired cognitive, motor, or hearing abilities or intake of central nervous system-active medication. Since superior sensorimotor synchronization (Chen et al., 2008; Repp, 2010; Krause et al., 2010a,b), as well as error correction (Repp, 2010) in musicians is well known, participants regularly practicing an instrument during the last 5 years prior to the study were excluded. To reduce the risk of infection with SARS-CoV-2, we additionally excluded participants with the respective disease symptoms and those having contact with people being positively tested for SARS-CoV-2 2 days prior to participation. To this end, each volunteer was asked for the relevant symptoms and personal contacts the day prior to participation by phone or email. Further exclusion criteria were clinically relevant depression as determined by the German version of the Beck’s Depression Inventory II (BDI II; Hautzinger et al., 2009) with test scores exceeding 9 points and general cognitive impairment by the German version of the Montreal Cognitive Assessment (MoCA; Bartusch and Zipper, 2004) with test scores below 26 points. Twenty-two participants (12 female) were assigned to the older group with a mean age of 53.4 ± 0.5 years (mean ± standard error of the mean, s.e.m.) ranging from 50 to 60 years. The younger group originally consisted of 29 participants, but data from one participant were excluded as the baseline synchronization was consistently two standard deviations larger than the group mean. The remaining 28 participants (18 female) were on average 24.0 ± 0.6 years old (range 20–36 years). All participants were right-handed as indicated by median EHI laterality quotients of 100 [interquartile range (IQR) = 8.75] in the older and 100 (IQR = 1.25) in the younger group (*Z* = −0.713, *p* = 0.476). Median BDI was 0 (IQR = 1) in both groups (*Z* = −0.230, *p* = 0.818), and none of the participants exceeded the cut-off value for depression of 9 points. Median MoCA was 30 (IQR = 0) in the younger group and 29.25 (IQR = 0.75) in the older group (*Z* = −1.534, *p* = 0.125) and none of the participants scored below the cut-off value for cognitive impairment of 26 points. The study was conducted in accordance with the latest version of the Declaration of Helsinki and was approved by the local ethics committee of the medical faculty of the Heinrich Heine University (study number 3347).

### Paradigm

Synchronization accuracy and error correction was determined by means of a sensorimotor synchronization task (for reviews see Repp, 2005; Repp and Su, 2013). To this end, a binaural tone (sine wave, duration 100 ms, 400 Hz) was presented *via* standard speakers with regular stimulus onset asynchronies (SOAs) of either 900 ms (sub-second condition) or 1,600 ms (supra-second condition). These SOAs were chosen as intervals between 200 and 1,800 ms can be reliably predicted by healthy participants while with larger intervals they tend to react to the pacing signal (Mates et al., 1994). Within a series of 10 auditory stimuli, the SOA randomly changed between the 4th and 7th tone in either positive or negative direction yielding longer or shorter intervals in a stepwise manner. Step-changes were either small (i.e., non-perceivable) or large (i.e., perceivable). The size of the step-change was determined by pilot data, where the threshold for identifying temporal deviations in an otherwise regular tone sequence was defined. Findings from the pilot study indicated asymmetrical detection thresholds with smaller step-changes being detected in positive as compared to negative deviations. The step-changes for each base interval are summarized in Table 1.

**Table 1:** Step-change depending on direction of phase-shift and base interval.

Sub-second: 900 ms SOA				Supra-second: 1,600 ms SOA			
Small phase-shifts		Large phase-shifts		Small phase-shifts		Large phase-shifts	
Negative	Positive	Negative	Positive	Negative	Positive	Negative	Positive
9%	6%	33%	24%	10%	7%	32%	24%
816 ms	954 ms	603 ms	1,116 ms	1,440 ms	1,712 ms	1,088 ms	1,984 ms

After each step-change, the SOA was kept constant until a series of ten taps was completed (i.e., at least for three subsequent taps) and then switched back to the initial SOA. At each position, step-changes were repeated five times in either direction resulting in 80 trials, for perceivable and non-perceivable step-changes, respectively.

The participants were instructed to synchronize their right index finger taps with the pacing signal as precisely as possible by tapping on a board (Elekta Neuromag^®^, Helsinki, Finland) thereby disrupting a photoelectric barrier that was connected to a standard Windows PC.

**Figure 1:**
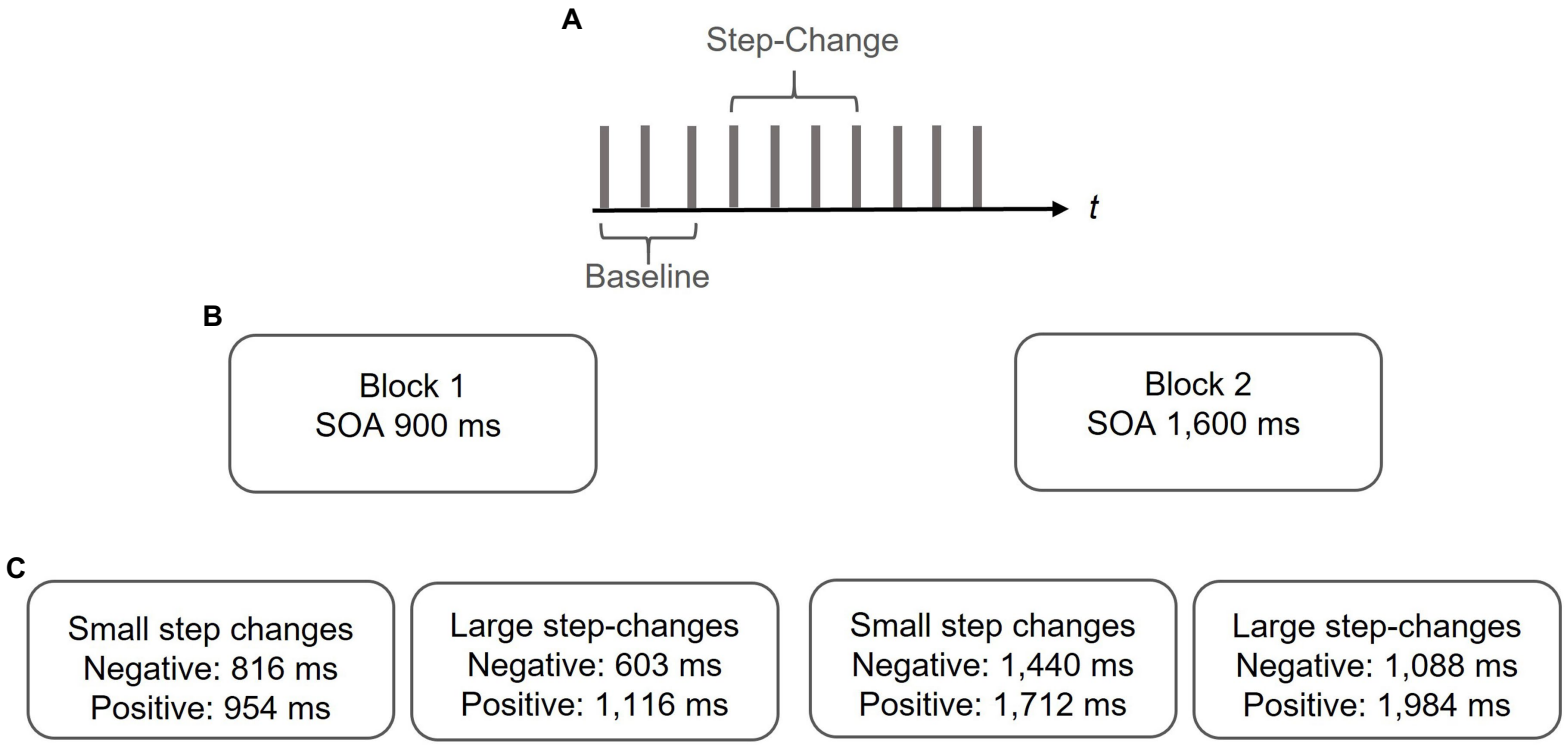
Paradigm. **(A)** In a series of 10 pacing signals a step-change was randomly interspersed between the 4th and 7th pacing signal. The 3 taps preceding the step-change served as a measure of baseline synchronization performance. **(B)** In two separate blocks baseline SOAs of either 900 or 1,600 ms were adopted. **(C)** Step-changes were either positive or negative and either small (i.e., non-perceivable) or large (i.e., perceivable). Data from a pilot study suggest asymmetrical detection thresholds with smaller deviations being detected in positive as compared to negative deviations. Step-changes were adapted with respect to those findings.

They were informed that the pacing signal may occasionally change the tempo. We decided to inform the participants about the tempo change as the data from the pilot study suggest that uninformed participants tended to omit the tap after the step-change, particularly in trials with large deviations. The paradigm is illustrated in Figure 1.

Reaction times were determined as a measure of processing speed (e.g., Buckhalt, 1991). To this end, the same pacing signal adopted for the synchronization task was used with SOAs of either 1,400, 2,400, or 3,200 ms. Each SOA was applied five times and the order was randomized. The participants were instructed to react as fast as possible when they heard the tone by tapping on the board with their right index finger.

The timing of tone presentation as well as the registration of tap onsets were realized by E-Prime 3 (Psychology Software Tools Inc., Sharpsburg, PA, United States). In each experimental condition, the tone was well audible and loudness was individually adjusted, if necessary.

### Design

**Figure 2:**
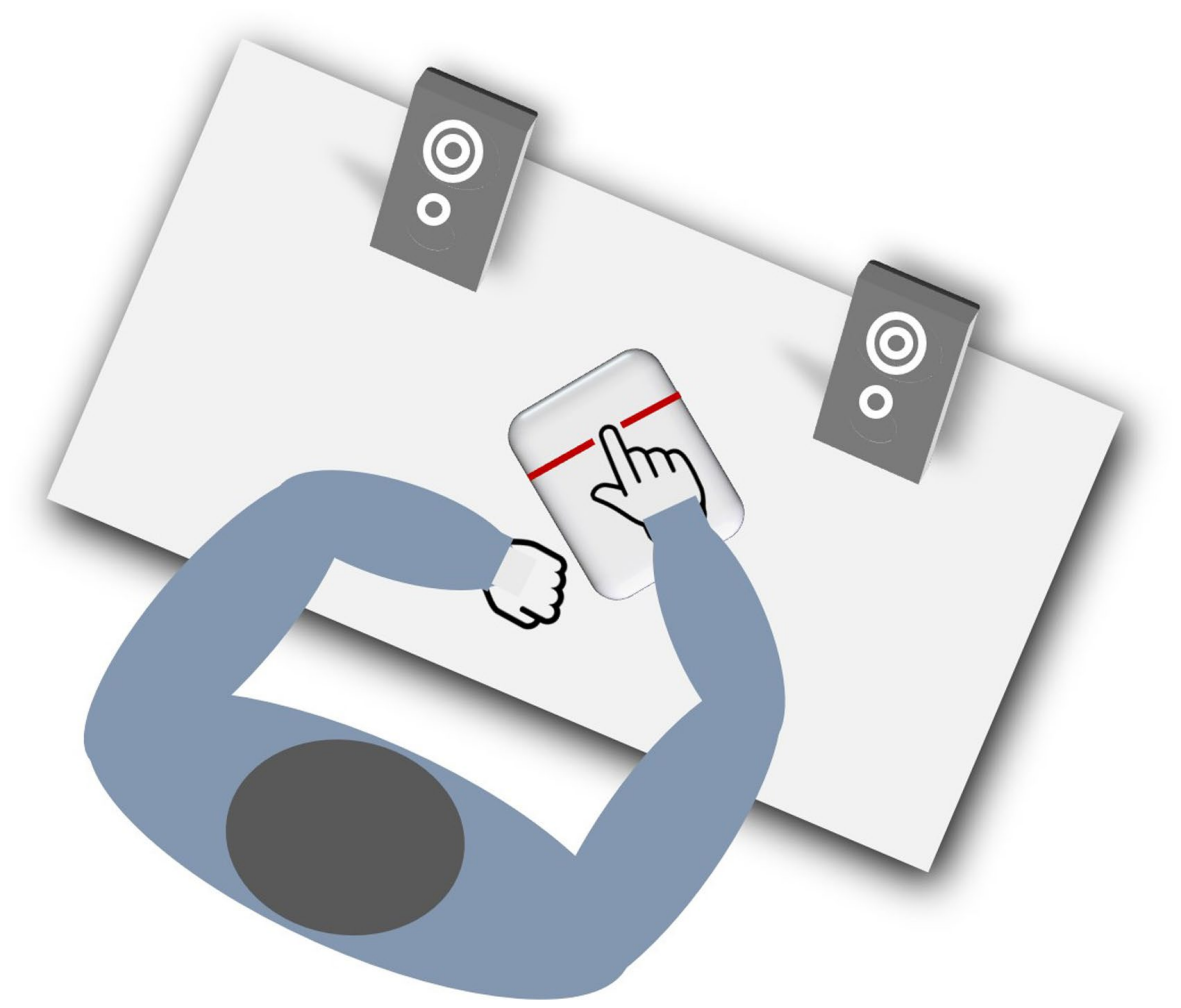
Experimental setup. The participants were seated at a table with standard speakers placed about 50 cm in front of them. Tap-onsets of the right index finger disrupted a photo-electric barrier mounted on a board (Elekta Neuromag^®^, Helsinki, Finland). Timing of tone presentation as well as registration of tap onsets were realized by E-Prime 3 (Psychology Software Tools Inc., Sharpsburg, PA, United States).

The study was conducted in a quiet and well-lit experimental room, and the participants were comfortably seated at a table with standard speakers placed about 50 cm in front of them (Figure 2).

The participants were naïve regarding the exact purpose of the study. To this end, they were instructed that the study aimed at investigating the effect of healthy aging on the temporally precise execution of movements during the general provision of information (i.e., prior to data acquisition). After finishing each experimental session, the exact intention of the study was clarified. We informed the participants that even in trials in which the metronome was apparently regular, small step-changes were interspersed and that the study intents at clarifying whether and to what extent, temporal irregularities (perceivable as well as non-perceivable) affected the temporal precision of the movements and whether this might be influenced by age. After obtaining written informed consent for study participation, the EHI, BDI II, and MoCA were completed, and subsequently reaction times were measured. The assessment of sensorimotor synchronization and error correction started with a test-trial allowing the participants to familiarize with the task and the apparatus. To this end, the same intervals and step-changes as in the experimental study were applied once. A training criterion was not adopted. Synchronization performance and error correction were then determined in two experimental blocks, separately for sub-and supra-second intervals. The block order was randomized and balanced across participants. Each block consisted of small and large step-changes of either direction. A break of individual length was provided between blocks. Each experimental session including information of the participants, completing the questionnaires, and obtaining consent took about 60 min. Each volunteer received 15 Euro as financial compensation for study participation.

### Analyses

For the three taps preceding each step-change the mean tap-to-tone asynchrony (i.e., the temporal distance between each tap-and tone-onsets) as well as the corresponding standard deviation were calculated as a measure of synchronization accuracy. Error correction was determined by calculating the phase correction response (PCR) (for a review see Repp and Su, 2013). To this end, the tap-to-tone asynchrony was determined at the position of the step-change (P) and the subsequent tap (P + 1). Then, the difference between both tap-to-tone asynchronies was calculated (asynchrony at P + 1 – asynchrony at P) as a percentage of the respective base interval and the sign was removed. The data were then averaged across trials separately for each experimental condition. Data two standard deviations below or above individual and group means were defined as outliers and removed from the analysis. At the group level, the data were replaced by the respective group mean. In individual data, less than 5% of all trials were removed according to this criterion and significant differences neither between groups nor between experimental conditions emerged (*p* > 0.09). At the group level, data from three younger and one older participant were removed and replaced by the respective group mean.

### Statistics

The data were controlled for Gaussian distribution my means of the Kolmogorov–Smirnov goodness-of-fit test. For group comparisons of reaction times, EHI, BDI, and MoCA Mann–Whitney-U-test was applied as the data were not normally distributed. Synchronization performance and PCR were analyzed using repeated measures analyses of variance (rmANOVA) with the between-subject factor *group* (older vs. younger) and within-subject factors *interval length* (sub-second vs. supra-second), *step-change direction* (positive vs. negative), and *size of step-change* (small vs. large). Statistics were conducted by means of IBM SPSS Statistics 27. *p*-values below 0.05 after correction for multiple comparisons by means of the sequential Bonferroni correction (Holm, 1979) were considered to be significant. Greenhouse–Geisser correction was applied whenever the sphericity assumption was violated.

## Results

**Figure 3:**
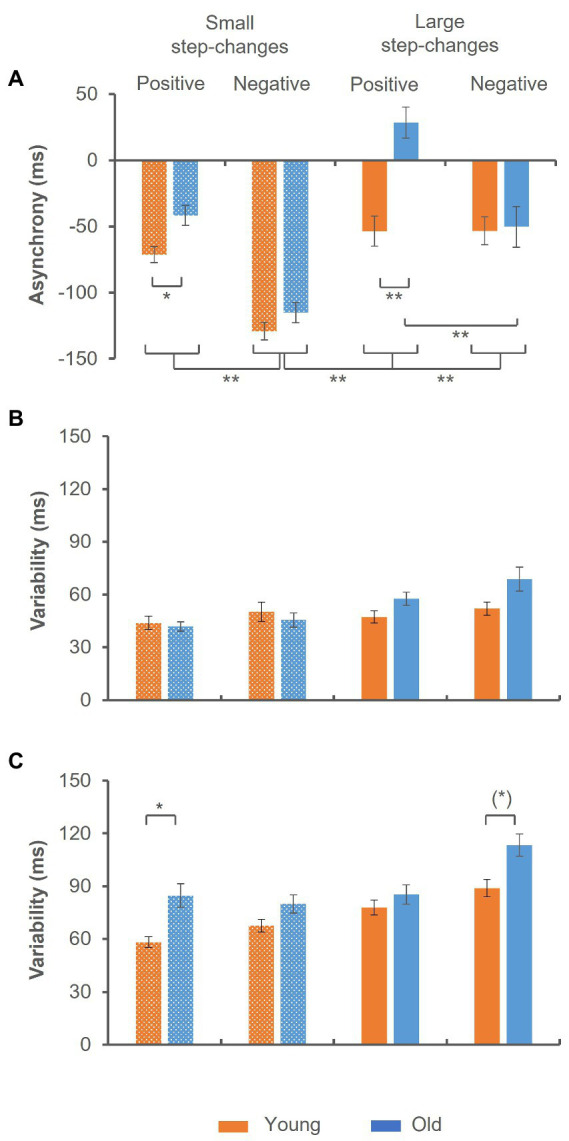
Synchronization accuracy in trials preceding the step-change. **(A)** Mean tap-to-tone asynchrony. Significant group differences emerged in trials preceding positive step-changes only. Prior to small step-changes the asynchrony was significantly smaller in the old group and changed to positive values prior to large step-changes. **(B,C)** Synchronization variability. While in trials with sub-second intervals no significant group-effect emerged **(B)**, variability was significantly larger in the old group in trials with supra-second intervals **(C)**. This was particularly evident in trials with small positive step-changes. A trend toward significantly larger variability in this group was found in trials with large negative step-changes. Error bars indicate the standard error of the mean (*) *p* ≤ 0.10; *p ≤ 0.05; **p ≤ 0.001.

Median reaction time was 193.57 ± 34.53 ms in the younger and 208.31 ± 42.67 ms in the older group (*Z* = −1.253, *p* = 0.210). The analysis of the mean tap-to-tone asynchrony suggested a significant *group* × *step-change direction* × *size of step-change* interaction [*F*(1,44) = 11.076, *p* = 0.002, *η_p_^2^* = 0.201], indicating significant group differences in trials with positive step-changes [*t*(48) = −4.411, *p* < 0.001], only. While in trials with small step-changes the tap-to-tone asynchrony was significantly smaller, but still negative in the older as compared to the younger group [*t*(48) = −2.872, *p* = 0.024], it turned to positive values in trials with large step-changes [*t*(48) = −0.149, *p* < 0.001]. In addition, independent of age the tap-to-tone asynchrony was significantly larger in trials preceding small negative step-changes as compared to all other conditions as indicated by a significant *step-change direction* × *size of step-change* interaction [*F*(1,44) = 5.170, *p* = 0.028, *η_p_^2^* = 0.105]. The data are summarized in Figure 3A.

The analysis of synchronization variability indicated significantly larger variability in trials with supra-second as compared to sub-second intervals as indicated by a significant main effect of *interval length* [*F*(1,44) = 173.347, *p* < 0.001, *η_p_^2^* = 0.798]. In addition, a significant *group* × *interval length* × *step-change direction* × *size of step-change* interaction emerged [*F*(1,44) = 4.578, *p* = 0.039, *η_p_^2^* = 0.094]. While in trials with sub-second intervals no significant difference between groups was found (Figure 3B), the variability was significantly larger in the older group in trials with supra-second intervals (Figure 3C). More precisely, variability was larger in trials preceding small positive step-changes [*t*(47) = −3.241, *p* = 0.024], and a trend toward significance after correction for multiple comparisons emerged in trials with large negative step-changes [*t*(46) = −2.874, *p* = 0.056].

**Figure 4:**
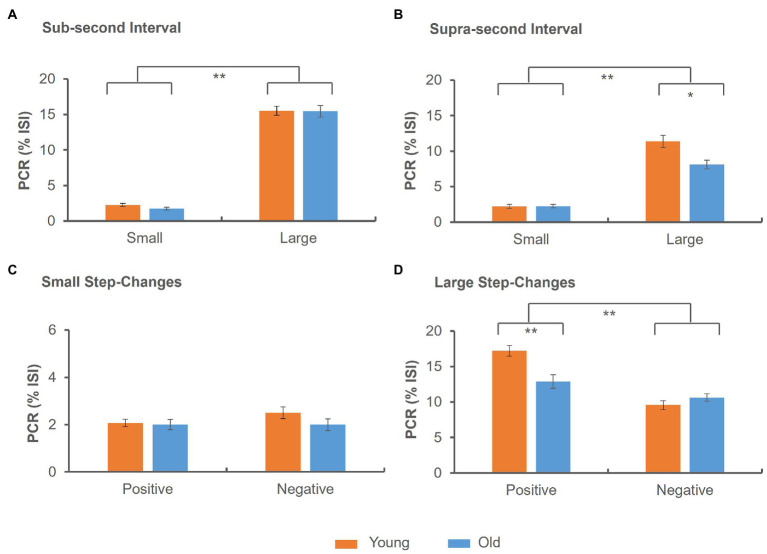
Phase correction response (PCR) in percent of the base SOA. **(A)** In trials with sub-second intervals no significant group differences emerged. **(B)** In trials with supra-second intervals the PCR was significantly smaller in the old as compared to the young group. **(C)** Small step-changes were not associated with significant group differences, **(D)** while large positive step-changes yielded significantly smaller PCR in the old as compared to the young group. Error bars indicate the standard error of the mean *p ≤ 0.05; **p ≤ 0.001.

The analysis of the PCR data indicated a significant main effect of *size of step-change* [*F*(1,41) = 696.175, *p* < 0.001, *η_p_^2^* = 0.944], indicating more pronounced PCR following large as compared to small step-changes (Figures 4A,B). In addition, a significant *group* × *size of step-change* × *interval length* interaction emerged [*F*(1,41) = 5.781, *p* = 0.021, *η_p_^2^* = 0.203], suggesting reduced PCR in the elderly in trials with supra-second intervals with large step-changes (Figure 4B). A significant *step-change direction x size of step-change* interaction [*F*(1,41) = 55.710, *p* < 0.001, *η_p_^2^* = 0.576] suggested significantly larger PCR in positive as compared to negative step changes in trials with large but not small step-changes (Figures 4C,D). A significant *group* × *step-change direction* × *size of step-change* interaction [*F*(1,41) = 10.451, *p* = 0.002, *η_p_^2^* = 0.203] indicated reduced PCR in the elderly in trials with large positive step-changes (Figure 4D), while the analysis of small step-changes did not suggest significant differences between age groups (Figure 4C).

## Discussion

The present study aimed at investigating age-related changes of error correction in a synchronization task adopting sub-and supra-second intervals. Error correction in the temporal domain was investigated by small (non-perceivable) as well as large (perceivable) step-changes which were interspersed in an otherwise regular tone sequence. We hypothesized a detrimental effect of age (i) on synchronization accuracy in trials with supra-second intervals and (ii) on error correction in trials with large step-changes due to age-related decline in the BG and the prefrontal cortex (Raz et al., 1995, 1997, 2005; Jernigan et al., 2001; Fjell et al., 2017); for reviews see (Seidler et al., 2010; Lockhart et al., 2014). A simple reaction time task served as control condition for the estimation of processing speed. In accordance with our hypotheses, reaction times did not significantly differ between groups, while reduced synchronization accuracy as well as error correction in the elderly was found in trials with supra-second intervals. The latter became evident in trials with large positive step-changes. The findings agree well with the notion of basically preserved sensorimotor processes operating in the sub-second range and age-related alterations within the BG and the prefrontal cortex interfering with sensorimotor timing in the supra-second range.

### Synchronization accuracy

Converging evidence exists that age is associated with a general slowing down of movements as indicated by the spontaneous (McAuley et al., 2006) as well as the fastest tapping rate (Turgeon et al., 2011; Turgeon and Wing, 2012); for a review see (Krampe, 2002). Noteworthy, such effects manifest late in life, namely in the seventh (Turgeon et al., 2011) or even ninth decade (Turgeon and Wing, 2012). In contrast to this, synchronization with simple isochronous rhythms do not appear to be affected by age (Krampe et al., 2005; Turgeon et al., 2011; Turgeon and Wing, 2012) although a slight increase of the synchronization error (i.e., the temporal difference between tap-and tone-onsets as a percentage of the inter-onset interval of the pacing signal) was reported in healthy participants above the age of 75 (Drewing et al., 2006).

The analysis of the present data suggests that synchronization performance was affected by the size and the direction of the step-change. In trials with small negative step-changes, larger negative asynchronies than in all other experimental conditions emerged. Although this result was surprising, it may be explained by asymmetrical error tolerance suggesting that participants are more sensitive in recognizing and correcting positive than negative asynchronies (for a review see Aschersleben, 2002). The data imply that negative step-changes may increase the tolerance for negative deviations. Interestingly, large step-changes did not result in a comparable effect. This might be due to the faster correction of perceived than non-perceived deviations (Praamstra et al., 2003; Bijsterbosch et al., 2011a,b), less affecting synchronization accuracy in subsequent trials.

Age-related differences emerged in trials preceding positive step-changes, only. Prior to small step-changes, the mean negative asynchrony was smaller in the elderly. While the asynchrony was still negative in this condition, it switched to positive asynchronies in trials with large step-changes. This result suggests that older participants tend to tolerate positive deviations more than the younger group. Moreover, the observation of positive asynchronies may indicate overcorrection in the elderly: Positive step-changes yielded larger negative asynchronies at the time point of the step-change as the interval became longer. Subsequently, the asynchrony returned to baseline level, which was on average negative. Since the older group switched to mean positive asynchronies, they seemed to overcompensate the preceding step-change.

The analysis of synchronization variability suggests larger variability in supra-second than sub-second trials, replicating previous findings (Drewing et al., 2006); for a review see (Krampe, 2002). Group differences were found in supra-second trials suggesting reduced synchronization accuracy in the elderly in tasks requiring more cognitive control. More precisely, larger variability emerged in trials with small positive step-changes, and a trend toward significantly larger variability was found in trials with large negative step-changes. The results indicate that deviations from the base interval interfere with task performance more strongly in the older than in the younger group, suggesting that the elderly tend to be more prone to fail on task requirements. Evidence for larger variability with increasing age in synchronization (Ivry and Keele, 1989); for a review see (Krampe, 2002) as well as continuation tasks in the sub-second range (Turgeon and Wing, 2012; Petter et al., 2016) exists. However, such decline has so far only been reported in higher age ranges, namely the seventh (Drewing et al., 2006; Petter et al., 2016) or even 9th decade of life (Turgeon and Wing, 2012). The present study adds to the existing literature by confirming that synchronization with supra-second intervals is already reduced in middle aged volunteers than for timing with sub-second intervals.

### Error correction

The analysis revealed more pronounced PCR following large than small step-changes independent of age. This result agrees well with previous findings, suggesting faster error correction in larger than smaller temporal deviations (Praamstra et al., 2003; Bijsterbosch et al., 2011a,b; Pollok et al., 2022). The present data suggest that age does interfere with error correction in the supra-second range, while no significant age-effect emerged in the sub-second range. The latter finding agrees well with previous data (Turgeon et al., 2011). Noteworthy, an age-related decline emerged in trials with large positive step-changes, suggesting reduced sensitivity for the detection and correction of positive deviations in the elderly. Participants are more sensitive in recognizing and correcting positive than negative asynchronies (Praamstra et al., 2003; Bijsterbosch et al., 2011a,b), for a review see (Aschersleben, 2002) suggesting asymmetrical error tolerance. This is particularly evident following large step-changes (Praamstra et al., 2003; Bijsterbosch et al., 2011b) and the present data support this finding. A previous study reported that the error-related negativity (ERN) is associated with large positive (i.e., + 50 ms), but neither with large negative (−50 ms) nor with small (+/−15 ms) phase shifts (Praamstra et al., 2003). The ERN likely originates in the ACC and was suggested to reflect an error detection mechanism (for an overview see Gehring et al., 2018), but might occur as a corrective behavioral response as well (Praamstra et al., 2003). For an ERN to show requires the integrity of the lateral PFC (Gehring and Knight, 2000), suggesting that successful error monitoring and correction depends on the functional interaction between both structures. More precisely, the PFC may provide the ACC with information necessary to distinguish between correct and incorrect responses (Gehring and Knight, 2000). The investigation of age-related changes of the ERN provides a heterogenous picture: On the one hand evidence exists that the ERN amplitude is attenuated in the elderly as compared to younger controls (e.g., Nieuwenhuis et al., 2002; Hoffmann and Falkenstein, 2011; Colino et al., 2017) and reduced metabolism in fronto-medial areas, including the ACC, has been shown with increasing age (Pardo et al., 2007). However, other data do not suggest changes across the adult lifespan (e.g., Hsieh and Fang, 2012; Niessen et al., 2017). The present data support the hypothesis that error correction during synchronization in the sub- and supra-second interval is differentially affected by age. While a detrimental effect was particularly evident in trials with large positive step-changes, we would propose that these changes might reflect diminished error detection and/or corrective responses in the elderly.

The DPFC has been related to attention and working memory processes (for a review refer to Rubia and Smith, 2004) and older participants can rely on less attentional resources than younger ones, affecting cognitive as well as motor performance (Voelcker-Rehage et al., 2006). Noteworthy, those age-related effects have been observed in dual-tasks, but not when the tasks were executed independently (Voelcker-Rehage et al., 2006). In the present study, intervals even in the supra-second condition were relatively short, and a single task was employed, suggesting that working memory and attentional demands were low. We thus would propose that different sub-regions within the DPFC subserving attentional and working memory functions on the one hand and timing functions on the other hand (Rubia and Smith, 2004) are already apparent as age-related decline of the latter in the fifth decade of life. Evidence supporting this hypothesis comes from a study indicating that reducing the excitability of the right DPFC by means of repetitive transcranial magnetic stimulation (rTMS) affects the reproduction of time intervals in the second, but not in the millisecond range (Jones et al., 2004). The age-related decline in error-correction shown in the present study agrees well with the observation of age-related atrophy within a BG-PFC circuit (Raz et al., 1997; Jernigan et al., 2001; Raz et al., 2005; Fjell et al., 2017); for reviews see (Seidler et al., 2010; Lockhart et al., 2014).

Taken together, the present data are consistent with the notion that (i) sensorimotor processes are efficiently operating until old age and (ii) age-related performance decline is linked to task demands (Li and Dinse, 2002; Krampe et al., 2005). The present data support the hypothesis that age-related decline in sensorimotor timing tasks in the supra-second range manifests as early as the age of 50 years in healthy adults.

## Limitations

In previous studies, step-changes between 10 ms (Praamstra et al., 2003; Doumas et al., 2005) and 18 ms (Bijsterbosch et al., 2011b) with baseline intervals of 500 or 600 ms were used to induce non-conscious error correction. We realize that the step-changes chosen here were much larger. Thus, we cannot exclude the possibility that the participants may have recognized the change at least in a subset of trials. Nevertheless, this would not explain the age-related decline of error correction in trials with large positive step-changes. Rather, one would expect that small step-changes are associated with a comparable age-effect.

There is ample evidence for an age-related decline in auditory-temporal processing (e.g., Fitzgibbons and Gordon-Salant, 2001; Fitzgibbons and Gordon-Salant, 2015), which was not explicitly tested here. We can therefore not exclude the possibility that the effects observed occur due to sensory decline. However, such age-related differences become evident in small deviations of subsequently presented tones, while in the present study impaired error correction was found in large deviations, only. Thus, even if auditory processing was impaired in the older participants, this effect should have been evident in trials with small rather than large deviations.

## Data availability statement

The raw data supporting the conclusions of this article will be made available by the authors, without undue reservation.

## Ethics statement

The studies involving human participants were reviewed and approved by the Ethics Committee of the Medical Faculty of the Heinrich Heine University, Duesseldorf.de. The patients/participants provided their written informed consent to participate in this study.

## Author contributions

BP, SK, and AH conceived and designed the experiment. AH performed the experiment. AH and BP analyzed the data. BP wrote the manuscript. VK and SK revised and edited the manuscript. All authors contributed to the article and approved the submitted version.

## Conflict of interest

The authors declare that the research was conducted in the absence of any commercial or financial relationships that could be construed as a potential conflict of interest.

## Publisher’s note

All claims expressed in this article are solely those of the authors and do not necessarily represent those of their affiliated organizations, or those of the publisher, the editors and the reviewers. Any product that may be evaluated in this article, or claim that may be made by its manufacturer, is not guaranteed or endorsed by the publisher.
